# Non-equilibrium molecular dynamics study of heat transfer parameters in two-dimensional Yukawa systems under uniform magnetic field

**DOI:** 10.1038/s41598-024-64866-z

**Published:** 2024-07-01

**Authors:** N. E. Djienbekov, N. Kh. Bastykova, T. S. Ramazanov, S. K. Kodanova

**Affiliations:** 1 Institute for Experimental and Theoretical Physics, Al-Farabi Kazakh National University, 71 Al-Farabi Ave., 050040 Almaty, Kazakhstan; 2 Institute of Applied Sciences and IT, 40-48 Shashkin Str., 050038 Almaty, Kazakhstan

**Keywords:** Magnetically confined plasmas, Thermodynamics

## Abstract

The present study explores the effect of a magnetic field on the thermal conductivity of two-dimensional (2D) Yukawa systems in a wide range of system parameters using the non-equilibrium molecular dynamic method (NEMD). We consider an external magnetic field with $\Omega =\omega _c/\omega _p\le 1$ (with $\Omega$ being the ratio of the cyclotron frequency to plasma frequency) and the coupling parameter values in the range $1\le \Gamma \le 100$ (with $\Gamma$ being the ratio of the Coulomb interaction energy at mean inter-particle distance to the thermal energy of particles). The results show that an external uniform magnetic field results in the reduction of the thermal conductivity at the considered values of the coupling parameter $\Gamma$. Additionally, we found that the effect of the magnetic field on thermal conduction is weaker at larger values of the system coupling parameter. To ensure that calculated results for the thermal conductivity are accurate and reliable, we performed a detailed investigation of the convergence of the results with respect to simulation parameters in NEMD with a strong external magnetic field. We believe that the presented results will serve as useful benchmark data for the theoretical models of (2D) Yukawa systems.

## Introduction

Since the seminal works by Landau^1^ and Spitzer^2^, the transport properties of charged systems have been actively investigated for both weakly and strongly coupled systems such as dusty plasmas^3,4^, warm dense matter^5^, hot dense plasmas^6^ etc. Despite the many ways of generating such systems of charged particles, the model system of charged particles interacting via screened Coulomb potential (Yukawa potential) often provides adequate and close descriptions of plasma properties^7–9^. This is particularly the case for dusty plasmas^10^, where charged dust particles form a 2D system of strongly coupled particles. Among other interesting physical phenomena, such systems provide a unique opportunity to study the effect of strong magnetic fields on the physical properties of strongly coupled plasmas^11–13^. Besides that, the study of transport characteristics of two-dimensional structures is relevant for applications in nanotechnology, microtechnology, and materials science.^14,15^. The 2D non-ideal structures are represented by such systems as dusty plasmas^7,11,12^, ions in extended lattice planes^16^, system of dipoles^17–19^, colloidal^20,21^ and polar molecules systems^22^.

As mentioned, an important model system on this topic is a non-ideal Yukawa system, which has been the focus of investigation by computer simulations in the last several decades. For example, using molecular dynamics techniques, computer simulations have been extensively used to investigate heat transfer in dense non-magnetized Yukawa systems under both equilibrium^23–25^ and non-equilibrium conditions^26,27^.

The calculation of thermal conductivity of 2D Yukawa systems using Green-Kubo relations within the standard equilibrium molecular dynamics (EMD) suffers from convergence issues due to slow decay of the relevant auto-correlation functions^24^. Donkó et al^24^ showed that the EMD approach is highly problematic for achieving convergence in the calculations of the thermal conductivity coefficient. In contrast, the nonequilibrium molecular dynamics (NEMD) method provides an efficient and physically transparent alternative. In the NEMD method, a heat flow is created between the hot and cold plates inducing a temperature gradient. This allows for the determination of the thermal conductivity coefficient in the system. This method is effective in overcoming the difficulties encountered with traditional EMD methods and has become a popular choice for the study of various transport properties of 2D systems^26,27^.

In Ref.^28^, the thermal conductivity of a three-dimensional (3D) strongly coupled and magnetized Yukawa system was examined to shed light on the effects of magnetic fields. The study found that the perpendicular heat transfer decreases under the influence of the magnetic field, while the parallel heat transfer even can increase.

To the best of our knowledge, there is no detailed and reliable report on the aspects of the numerical convergence of the NEMD method for the computation of the thermal conductivity of 2D Yukawa systems under an external magnetic field. Such a detailed study is important for obtaining reliable data on the thermal conductivity coefficients of 2D Yukawa systems under an external magnetic field. Therefore, in this work, we presented a detailed NEMD-based study of the thermal conductivity of the two-dimensional strongly coupled Yukawa system under the influence of a magnetic field. This provides valuable insights and a more comprehensive understanding of the thermal conductivity in weakly and strongly magnetized charged 2D systems.

This study aims to explore the impact of a magnetic field on the thermal conductivity of two-dimensional (2D) Yukawa systems. To achieve this goal, we utilized the nonequilibrium molecular dynamics method to perform calculations for a wide range of system parameters. First, we provide details of the used computation methods in Sec. 1. After that, the simulation results with and without magnetic fields are reported in Sec. 2. We conclude the paper by summarizing our findings.

## Computational method and simulation parameters

Let us delve into the simulation methodology employed for determining thermal conductivity in a magnetized Yukawa system. Specifically, the nonequilibrium molecular dynamics (NEMD) method^26^ was utilized for this purpose. The simulation was conducted within a square box with periodic boundary conditions. The pair interaction potential between particles is set to the Yukawa potential:

1 $$\begin{aligned} \beta V(r)=\frac{\Gamma }{r}exp(-\kappa r), \end{aligned}$$

where *r* is in the units of the mean inter-particle distance, $\beta =1/(k_BT)$ is the inverse value of a thermal energy, $\kappa = k_s a$ is screening parameter, $k_s$ is the inverse screening length and $\Gamma = \frac{Q^2}{4\pi \varepsilon _0 a k_B T}$ is the coupling parameter.

Using molecular dynamics, we simulate a system comprising 1600 particles that are confined within a square simulation box under periodic boundary conditions. The length of the simulation box, denoted by *L*, is determined by the number of particles via the relation $L/a = \sqrt{\pi N}$, where *a* represents the average inter-particle distance. Throughout the simulations, we measure lengths in units of *a*, and time in units of the inverse plasma frequency $1/\omega _p = \left( \frac{Q^2}{2\pi \varepsilon _0 ma^3}\right) ^{-1/2}$, where *Q* denotes the electric charge, $\epsilon _0$ is the permittivity of free space, and *m* is the particle mass. The unit of energy is taken to be $\varepsilon = Q^2/(4\pi \varepsilon _0a)$.

The system under consideration is governed by two dimensionless parameters, namely the coupling parameter ($\Gamma$) and the screening parameter ($\kappa$). The thermal conductivity of the system is expressed in the units of $\lambda _0 = mn\omega _p a^2 k_B$, where $k_B$ is Boltzmann’s constant and $n = (\pi a^2)^{-1}$ is the number density of particles. The velocity values of the system are expressed in the units of $v_0 = a\omega _p$. $T^{*}=k_BT/\varepsilon$ and ${\frac{\partial T^{*}}{\partial y^{*}}}={\frac{\partial T^{*}}{\partial (y/a)}}$ are dimensionless temperature and temperature gradient, respectively.

### Equations of motion used to include a homogeneous magnetic field

The Velocity Verlet algorithm is a widely used numerical method for solving the equations of motion of particles in the presence of a homogeneous magnetic field, as described by Spreiter et al.^29^ in their work on classical molecular dynamics. This algorithm is particularly useful for simulating complex systems of charged particles, such as plasmas, where the magnetic force plays a crucial role in determining their dynamics.

The equations of motion for a charged particle in an external homogeneous magnetic field are driven by the Lorentz force law. In the case of a uniform magnetic field, the force is always perpendicular to the velocity of a particle, causing it to move in a circular path. The magnitude of the force is proportional to the charge and velocity of the particle, as well as the strength of the magnetic field. Following Spreiter et al.^29^, the equations governing coordinates read:

2 $$\begin{aligned} x(t + \Delta t)= & {} x(t) + \frac{1}{\Omega } \left[ v_x(t)\sin (\Omega \Delta t) - v_y(t)C(\Omega \Delta t)\right] + \frac{1}{\Omega ^2} \left[ -a_x^c(t)C(\Omega \Delta t) - a_y^c(t)S(\Omega \Delta t)\right] + O((\Delta t)^3), \end{aligned}$$

3 $$\begin{aligned} y(t + \Delta t)= & {} \text {same as Eq. 2 with}\ (x \leftrightarrow y, \Omega \leftrightarrow -\Omega ). \end{aligned}$$

The velocity components are defined as

4 $$\begin{aligned} v_x(t+\Delta t)= & {} v_x(t)\cos (\Omega \Delta t) + v_y(t)\sin (\Omega \Delta t) + \frac{1}{\Omega } \left[ -a_y^c(t)C(\Omega \Delta t) + a_x^c(t)\sin (\Omega \Delta t)\right] \nonumber \\{} & {} + \frac{1}{\Omega ^2} \left[ -\frac{a_x^c(t+\Delta t)-a_x^c(t)}{\Delta t}C(\Omega \Delta t) -\frac{a_y^c(t+\Delta t)-a_y^c(t)}{\Delta t}S(\Omega \Delta t)\right] + O((\Delta t)^3), \end{aligned}$$

5 $$\begin{aligned} v_y(t+\Delta t)= & {} \text {same as Eq. 7 with}\ (x \leftrightarrow y, \Omega \leftrightarrow -\Omega ), \end{aligned}$$

where

6 $$\begin{aligned} S(\Omega \Delta t) = \sin (\Omega \Delta t) - \Omega \Delta t, \end{aligned}$$

and

7 $$\begin{aligned} C(\Omega \Delta t) = \cos (\Omega \Delta t) - 1. \end{aligned}$$

**Figure 1:**
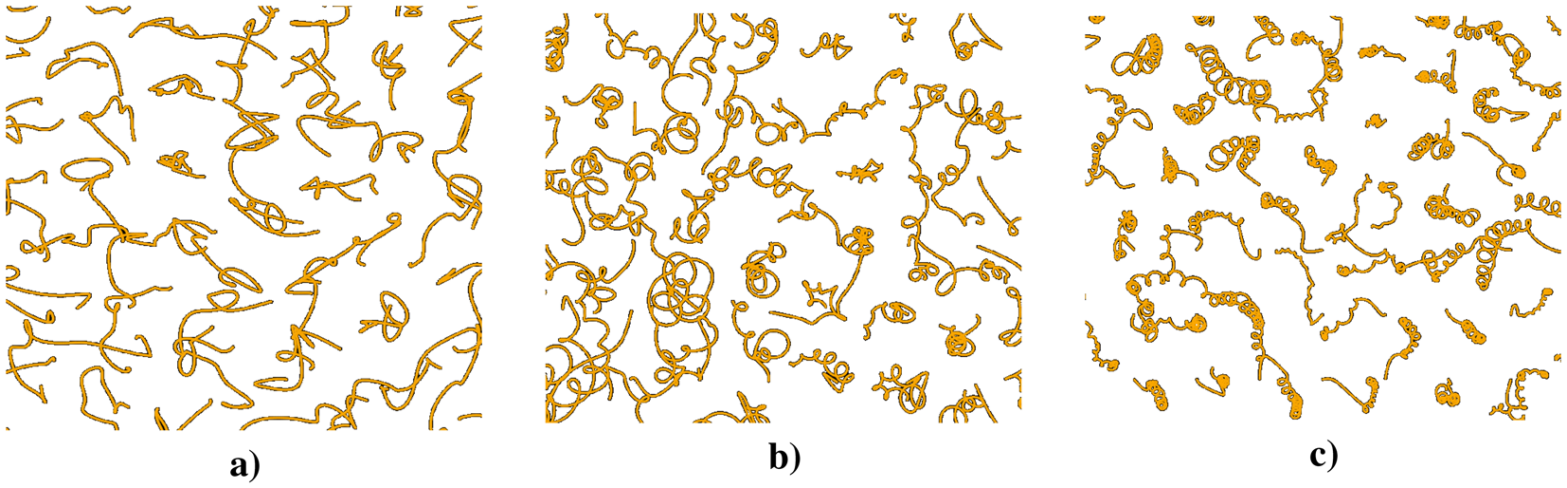
Trajectories of the particles at $\Gamma = 50$, $\kappa = 2$ for (**a**) $\Omega = 0$, (**b**) $\Omega = 0.5$, and (**c**) $\Omega = 1.0$.

We consider the magnetic field to be directed perpendicular to the 2D plane on which particles are confined. For the equilibrium case, we illustrate a characteristic pattern of the motion of the particles with $\Gamma =50$ and $\kappa =2$ and without the effect of the magnetic field in Fig. 1a. For given parameters of the inverse screening length and coupling parameter, the particles interact with each other through the Yukawa potential, which leads to the chaotic motion of the particles as in a liquid. The interaction of the particles with each other creates complex oscillations and dynamics in the plasma system. Trajectories of particles under an external magnetic field with $\Omega = 0.5$ are illustrated in Fig. 1b. In this case, it can be observed that the trajectories of the particles become curved and begin to twist around the magnetic field lines. Trajectories of particles in a strong magnetic field with $\Omega = 1$ are demonstrated in Fig. 1c. At this value of the magnetic field, the Larmorian twisting of the particles becomes even more pronounced and the particle trajectories begin to take on complex and well pronounced spiral shapes.

### The NEMD method for generating temperature gradient

**Figure 2:**
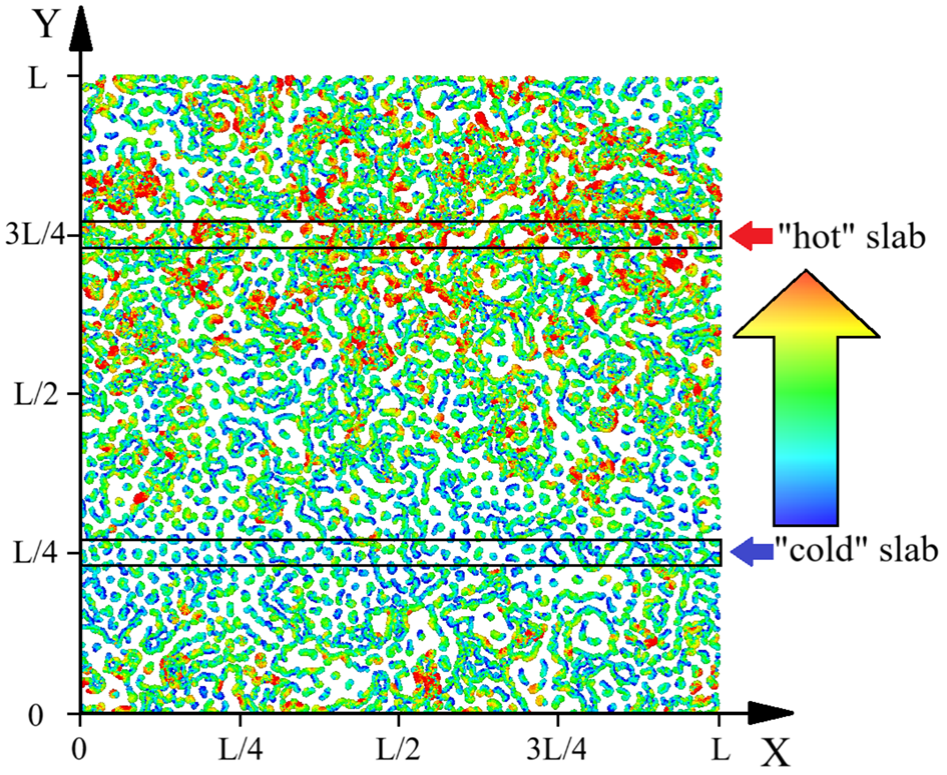
Color-coded particle trajectories at $\Gamma = 50$, $\kappa = 2$, $\Omega = 1$, where the absolute value of the velocity increases from blue (relatively cold particles) to red (relatively hot particles).

In contrast to equilibrium methods, non-equilibrium methods allow the system to be brought into a state that is close to the conditions with the macroscopic directional fluxes of mass and energy. To create a directed heat conduction, we use the Müller-Plate method^26^. We divide our two-dimensional system into plates parallel to the x-axis. To calculate heat conduction, we need to create an energy flux in the system while preserving the total energy. To achieve this, we select two slabs at heights of $y=(1/4)L$ and $y=(3/4)L$, which illustrated on Fig. 2. According to Müller-Plate method, at certain MD steps, in one slab we find the particle with the highest velocity modulus, while in the other, we find the particle with the lowest velocity modulus. We swap their impulses, changing the velocity modules. Repeating this periodically forms a gradient of temperature between slabs. The temperature of the *k*-th plate is determined by this formula:

8 $$\begin{aligned} T_k = \frac{1}{N_k k_B} \sum \limits _{i \in k}^{N_k} m_i v_{i}^2, \end{aligned}$$

where $N_k$ is the number of particles in the k-th slab. The dependence of the temperature between slabs on the distance between slabs can be regulated to be nearly linear. Using the latter fact, we can determine the temperature gradient and calculate the thermal conductivity.

As we know, the flux of thermal energy is directly proportional to the temperature gradient, according to Fourier’s law:

9 $$\begin{aligned} A_E = -\lambda \frac{\partial T}{\partial y}. \end{aligned}$$

On the other hand, the flux of thermal energy can be calculated if the total value of the transferred thermal energy $\Delta E$ in a given period *t* is known^26^:

10 $$\begin{aligned} A_E = \frac{\Delta E}{2Lt}, \end{aligned}$$

where $\Delta E = \sum \limits _{\text {transfers}} \frac{m}{2}(v^2_h - v^2_c)$, $v_h$—speed of “hottest” particle, $v_c$—speed of “coldest” particle. From these two formulas for heat flow, one finds an expression for thermal conductivity^26^:

11 $$\begin{aligned} \lambda = -\frac{\Delta E}{2Lt \frac{\partial T}{\partial y}} \end{aligned}$$

We illustrated the heating and cooling of slabs in Fig. 2. The image displays the trajectories of particles in a system with a coupling parameter of $\Gamma = 50$, a screening parameter of $\kappa = 2$, and a magnetic field value of $\Omega = 1$. The trajectories are color-coded to indicate the velocities of the particles, with blue representing low velocity and red representing high velocity. The color gradient also provides information about the temperature gradient in the system, with warmer areas corresponding to higher average particle velocity, indicating higher kinetic energy and temperature.

## Results

### NEMD simulations without magnetic field

The main focus of this paper is the study of the effect of an external uniform magnetic field on the heat conductivity coefficient of the 2D system of charged Yukawa liquids. To perform such analysis adequately, naturally, we need to start first from the reference state represented by the case without a magnetic field.

**Figure 3:**
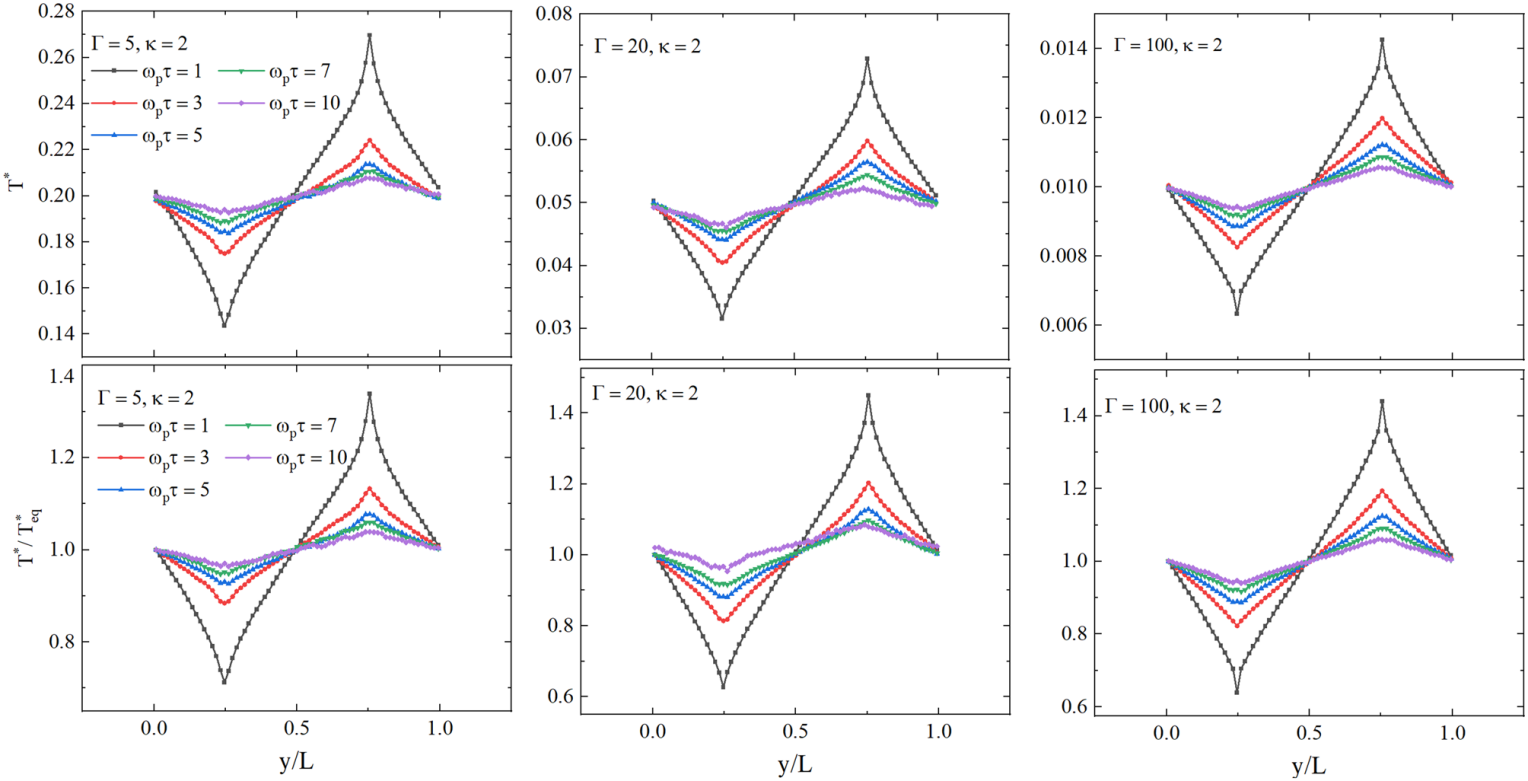
Temperature in the system for $\Gamma =5$, 20, 100, and $\kappa = 2$ at different values of the velocity swap period in the NEMD calculations. The upper graphs show the dependence of temperature on the distance along the *y* component in units of *L*, the lower graphs show the dependence of the temperature expressed in units of the unperturbed nonequilibrium system temperature value on the distance along the *y* component in units of *L*.

To obtain reliable results using the NEMD method, we need to study the dependence of the calculated thermal conductivity coefficient on the velocity replacement period in the Müller-Plate method. To calculate the thermal conductivity at given system parameters, it is important to have the coupling parameter and temperature in the system weakly altered. This can be problematic if the perturbation of the system in the NEMD simulations is too strong and, as a result, the generated anisotropy of the system parameters is significant. In the considered case, as one might expect, a smaller velocity swap period creates a larger spread of temperature values in the system, which also leads to uncertainty in the coupling parameter. This is shown in Fig. 3, where the temperature distribution between heated and cooled slabs are presented for $\Gamma =5$ (left), $\Gamma =20$ (middle), $\Gamma =100$ (right), and at different values of the velocity swap period $1 \le \omega _p\tau \le 10$. From Fig. 3, we clearly observe that the increase in the swap period $\tau$ results in a smaller deviation of the temperature distribution from the reference temperature of the undisturbed system.

An adequate value of the thermal conductivity coefficient requires that the perturbation of the system is weak, i.e, $T^{*}(y)/T^{*}_{{\text{eq}}}\simeq 1$, where $T^{*}(\textbf{r})$ is the local value of the temperature between heated and cooled slabs and $T^{*}_{\text{eq}}$ is the temperature of the equilibrium unperturbed system. From Fig. 3 (second line), we see that for $\Gamma =5$ we have $\delta T^{*}(y)=T^{*}(y)-T^{*}_{\text{eq}}<0.1 T^{*}_{\text{eq}}$ at $\omega _p\tau \ge 5$. For $\Gamma =20$ and $\Gamma =100$, we have $\delta T^{*}(y)<0.1 T^{*}_{\text{eq}}$ at $\omega _p\tau \ge 7$. The right panel of first line of Fig. 3 for $\Gamma = 100$ corresponds to the case when inter-particle interactions dominate over their kinetic energy. This regime leads to a more ordered system. This order limits the spatial variation of the kinetic energy and, consequently, the temperature deviation from the equilibrium value of it. Thus, as $\Gamma$ increases, an anisotropy in the temperature distribution decreases. This behavior contrasts with the case $\Gamma = 5$ (Fig. 3 left panel, first line), where particles have more mobility, leading to larger temperature gradients.

**Figure 4:**
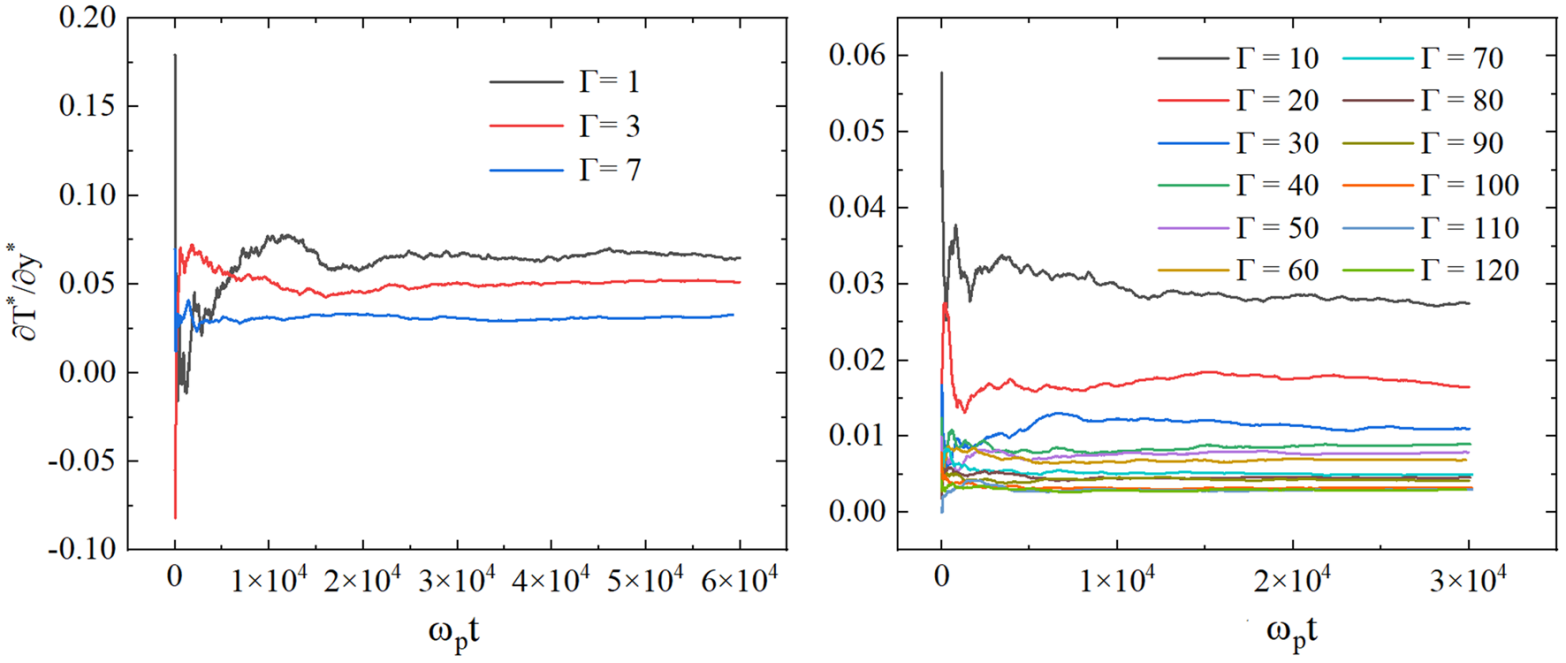
The dependence of the temperature gradient on time for different $\Gamma$ values and $\kappa = 2$.

Let’s discuss the time when the steady state is achieved. To account for the time dependence of the temperature gradient, we have taken great care to ensure that only the part of the gradient that has achieved its state of “saturation” is considered in our calculations. Specifically, we have allowed the system to reach a steady state, waiting for the period 30,000 $\omega _p t$ when $\Gamma \ge 10$ (see the examples in the left panel of Fig. 4) and 45,000 $\omega _p t$ when $\Gamma < 10$ (see the examples in the right panel of Fig. 4). For small $\Gamma$, it takes more time to establish a steady state of the system because the thermal chaotic motion of particles begins to dominate over the potential interaction. To further enhance the precision of our results, we have performed 50 independent calculations for each data point, and subsequently computed the average of the outcomes.

**Figure 5:**
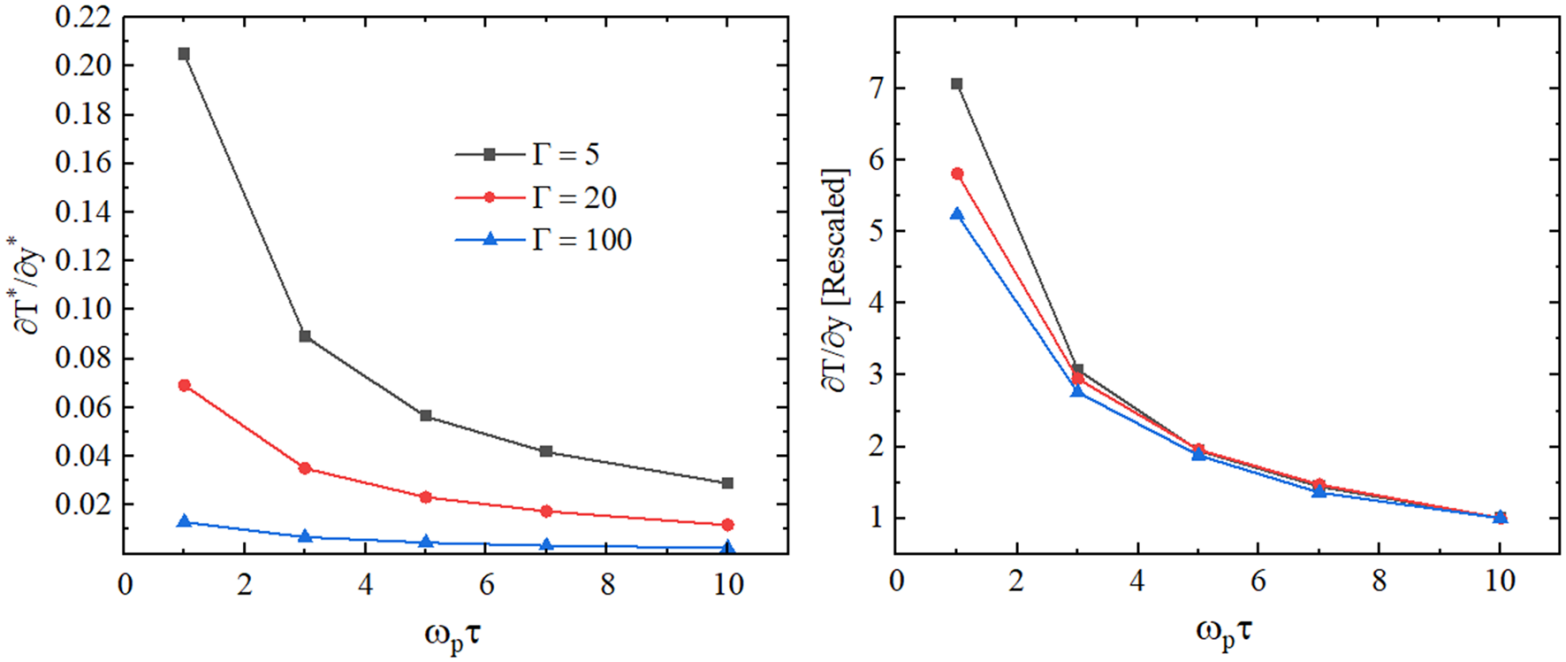
Dependence of the temperature gradient on the velocity swap period $\tau$ for $\Gamma =5, 20, 100$ and $\kappa = 2$. The right panel shows the magnitude of the temperature gradient divided by the result computed by setting $\tau =10$.

In Fig. 5, we show the dependence of the temperature gradient between heated and cooled slabs on the parameter $\tau$. From Fig. 5, we see that the increase in the velocity swap period $\tau$ results in a decrease in the magnitude of the temperature gradient between slabs. In other words, a shorter period $\tau$ of the velocity swap leads to a steeper temperature gradient within the system. The right panel of Fig. 5 shows the temperature gradient values normalized with respect to the value obtained at $\tau =10$. This normalization is performed by dividing the measured temperature gradient for each $\tau$ by the value of the gradient at $\tau =10$. This allows us to better visualize the change rate in the temperature gradient with the increase in $\tau$. Interestingly, we observe that the dependence of the rescaled temperature gradient on $\tau$ becomes nearly independent of $\Gamma$ at $\omega _p\tau \ge 5$.

**Figure 6:**
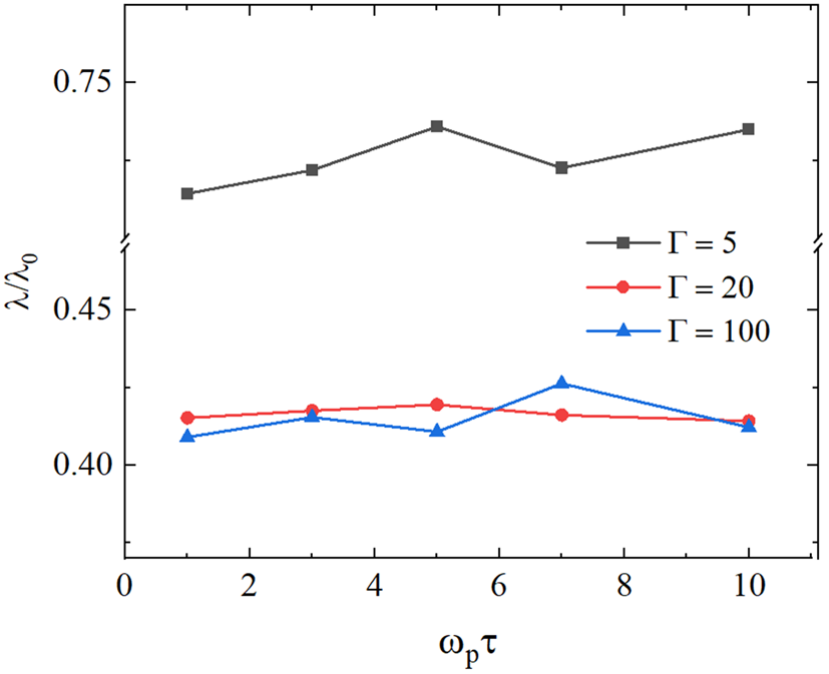
Thermal conductivity coefficient of the 2D Yuakwa system without an external magnetic field effect as a function of the velocity swap period $\tau$ for $\Gamma =5, 20, 100$ and $\kappa = 2$.

**Figure 7:**
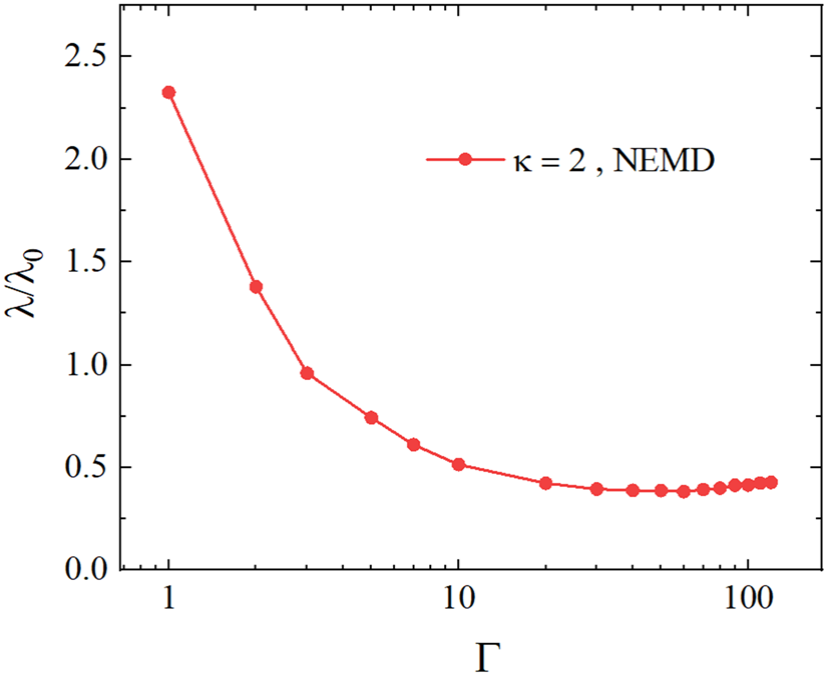
Dependence of thermal conductivity on the coupling parameter, in the absence of magnetic field.

Overall, it is clear that by choosing an optimal replacement period, we can minimize uncertainty in the coupling parameter and temperature, and obtain reliable thermal conductivity values. The thermal conductivity values for $\Gamma =5$, $\Gamma =20$, and $\Gamma =100$ at different values of the parameter $\tau$ are shown in Fig. 6. From Fig. 6, we can see that there is no substantial change in the thermal conductivity coefficient at the considered $\tau$ values. For $\Gamma =100$, we have the change in the thermal conductivity within $\delta \lambda =\pm 0.05 \lambda _0$ due to the variation in $\tau$. For $\Gamma =20$, we have $\delta \lambda =\pm 0.01 \lambda _0$. For $\Gamma =5$, we have $\delta \lambda =\pm 0.025 \lambda _0$. The close values of the thermal conductivity at $\Gamma =20$ and $\Gamma =100$ are due to the non-monotonic dependence of the thermal conductivity on $\Gamma$. Figure 7 shows the thermal conductivity coefficient computed without a magnetic field for different $\Gamma$. At low $\Gamma$ values, the thermal conductivity of the system is high because the weak interaction between particles allows efficient transfer of kinetic energy through frequent collisions. However, as $\Gamma$ increases, the interactions between particles become stronger, which reduces their mobility and hinders direct energy transfer, leading to a drop in thermal conductivity. At high $\Gamma$, where potential interactions dominate, heat transfer is realized through collective potential interaction, but this mechanism is less efficient compared to direct kinetic transfer, which explains the slow increase of thermal conductivity with a further increase of $\Gamma$.

### NEMD simulations with an external magnetic field

**Figure 8:**
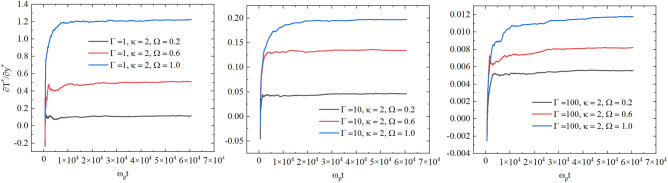
The dependence of the temperature gradient on time for $\Gamma = 1, 10, 100$, $\kappa = 2$, and $\Omega = 0.2 , 0.6, 1.0$.

To ensure the accuracy of thermal conductivity measurements in the presence of a magnetic field, we have performed accurate tests of the convergence of the results with respect to simulation parameters. In Fig. 8, we show the dependence of the temperature gradient on time for $\Gamma = 1$ (left panel), $\Gamma = 10$ (middle panel), and $\Gamma = 100$ (right panel). The results in Fig. 8 are computed at $\kappa = 2$, $\Omega = 0.2$, 0.6, and 1.0. From Fig. 8, we see that the period 45,000 $\omega t$ is enough time for the system to reach a steady state at all considered parameters. We note from Fig. 8 that the magnetic field reduces the amplitude of fluctuations in the temperature gradient values around the mean steady-state value. In addition, as the strength of the magnetic field increases, the value of the temperature gradient also increases.

**Figure 9:**
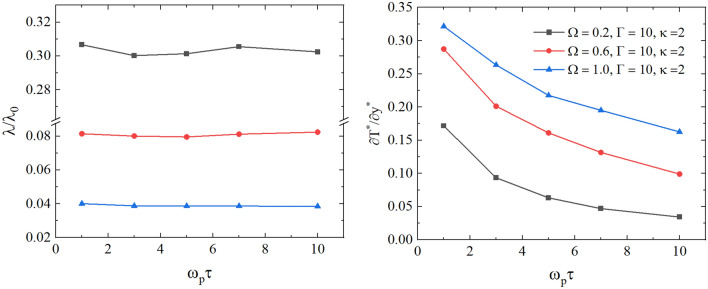
Left: the dependence of the thermal conductivity coefficient on the parameter $\tau$. Right: the dependence of the temperature gradient on the velocity swap period $\tau$. The results are for $\Gamma = 10$, $\kappa = 2$, and $\Omega =0.2 , 0.6, 1.0$.

In our calculations, to achieve high accuracy, we have performed averaging over 50 independent NEMD calculations of the thermal conductivity coefficient. We also tested the dependence of the thermal conductivity on the velocity swap period in the presence of the external magnetic field. In the left panel of Fig. 9, we show the dependence of the thermal conductivity coefficient on the velocity swap period $\tau$ at $1\le \tau \omega _p\le 10$ for parameters $\Gamma =10$, $\kappa =2$, and $\Omega =0.2$, $\Omega =0.6$, and $\Omega =1.0$. From the left panel of Fig. 9, one can see that the thermal conductivity coefficient is nearly independent of $\tau$ for $1\le \tau \le 10$ under the considered external uniform magnetic field. This does not mean that the temperature gradient is not sensitive to the parameter $\tau$ in our NEMD simulations. In the right panel of Fig. 9, we show the dependence of the magnitude of the temperature gradient on the velocity swap period $\tau$ for parameters $\Gamma =10$, $\kappa =2$, and $\Omega =0.2$, $\Omega =0.6$, and $\Omega =1.0$. As expected, we observe from the right panel of Fig. 9 that the increase in $\tau$ results in smaller values of the temperature difference between heated and cooled slabs. Therefore, for considering $\tau$ values, we see that the energy exchange $\delta E$ and the temperature difference between slabs change with the variation of $\tau$ in such a way that the thermal conductivity remains approximately unaffected. At $\tau > 3$, the variation of the thermal conductivity does not exceed $2\%$ at all considered parameters.

We note from the left panel of Fig. 9 that the thermal conductivity coefficient is smaller at larger values of the external magnetic field strength (i.e, $\Omega$). The rotation of the particles around the magnetic lines (as illustrated in Fig. 1) results in a reduction in the energy transfer between particles. As a result, the temperature gradient becomes more pronounced (see the right panel of Fig. 9), and the system becomes less efficient in transferring energy.

### Thermal conductivity coefficient

After properly identifying the simulation parameters of the NEMD for an adequate computation of the thermal conductivity, we have performed calculations of the thermal conductivity coefficient for $\Gamma =1$, $\Gamma =10$, $\Gamma =50$, and $\Gamma =100$ at $\Omega =0.0$, $\Omega =0.2$, $\Omega =0.4$, $\Omega =0.6$, and $\Omega =1.0$.

**Figure 10:**
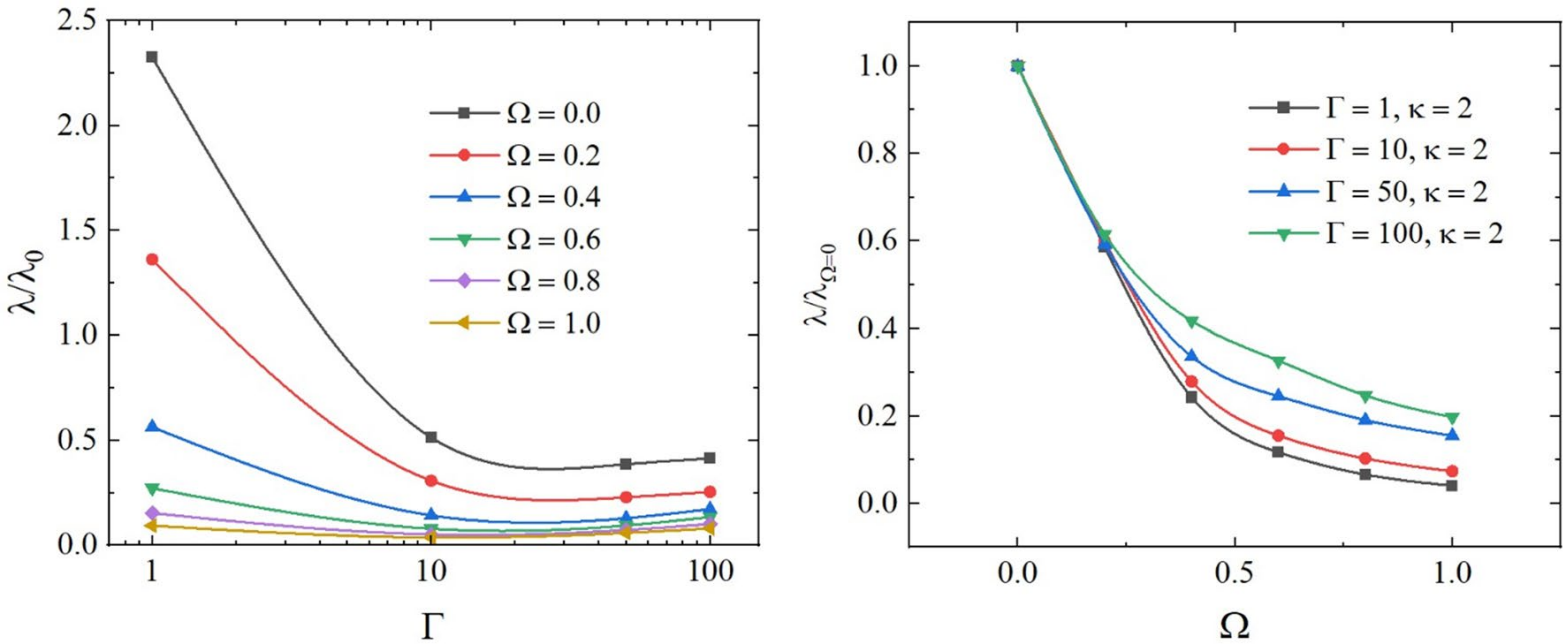
Left: The dependence of the thermal conductivity coefficient on the coupling parameter $\Gamma$ for different values of $\Omega$. Right: the dependence of the ratio $\lambda /\lambda _{\Omega =0}$ on the strength of the external magnetic field defined by $\Omega$.

In the left panel of Fig. 10, we illustrate the dependence of the thermal conductivity coefficient on $\Gamma$ at different values of the magnitude of the external magnetic field. Our results show that the heat transfer coefficient decreases as the magnetic field increases. Therefore, the applied magnetic field reduces the system’s thermal conductivity. This observation is consistent with the fact that the magnetic field restricts the motion of charged particles, leading to a decrease in thermal conductivity.

From the left panel of Fig. 10, we see the well-known dependence of the thermal conductivity on the coupling parameter $\Gamma$^24^: the decrease in $\Gamma$ at $\Gamma <10$ (for $\kappa =2$) leads to the increase in the thermal conductivity $\lambda$ since the kinetic effects have a dominant contribution to $\lambda$ and the increase in $\Gamma$ at $\Gamma \gtrsim 50$ (for $\kappa =2$) results in the increase in the thermal conductivity $\lambda$ because the inter-particle correlations have the dominant role in defining $\lambda$ values. A uniform external magnetic field does not change this qualitative picture of the dependence on $\Gamma$. However, the increase in the magnitude of the external magnetic field strength results in a significant decrease in the thermal conductivity. For example, at $\Omega =1$, we have a decrease about of one order of magnitude in $\lambda$ compared to the case without a magnetic field (see Table 1).

In order to have more insight into the effect of the variation of $\Omega$ on the thermal conductivity coefficient, we show the ratio $\lambda /\lambda _{\Omega =0}$ as a function of $\Omega$ in the right panel of Fig. 10, where $\lambda _{\Omega =0}$ is the thermal conductivity without magnetic field. We observed that at lower values of the plasma coupling parameter, the reduction in the thermal conductivity compared to $\lambda _{\Omega =0}$ is stronger with the increase in the magnitude of the magnetic field strength at $\Omega >0.2$.

Overall, our analysis highlights the complex interplay between the effect of the magnetic field and plasma non-ideality in determining the thermal conductivity coefficient. Our findings suggest that the rate of thermal conductivity decrease is not solely determined by the magnetic field strength but also by the intensity of interactions within the plasma. The effect of the magnetic field on the thermal conductivity reduces with the increase in the coupling parameter. This can be understood by recalling that stronger repulsion between particles results in stronger localization of particles in corresponding local minima on the potential energy surface^30^, which reduces the impact of the magnetic field-induced localization on the thermal conductivity.

Our results of the NEMD calculations of the thermal conductivity of the 2D Yuaka system are summarized in Table 1.

**Table 1:** Thermal conductivity coefficient for different values of $\Gamma$ and $\Omega$ for $\kappa = 2$.

$\Gamma$	$\Omega = 0.0$	$\Omega = 0.2$	$\Omega = 0.4$	$\Omega = 0.6$	$\Omega = 0.8$	$\Omega = 1.0$
1	2.326	1.362	0.565	0.273	0.155	0.095
10	0.515	0.309	0.1438	0.08	0.053	0.038
50	0.388	0.23	0.130	0.095	0.0741	0.060
100	0.415	0.255	0.173	0.136	0.103	0.082

The results were obtained setting the the velocity swap period to $\tau =10$ in the NEMD simulations.

## Conclusions

We analyzed the thermal conductivity coefficient of the two-dimensional Yukawa system under a uniform external magnetic field at different values of the coupling parameter using the non-equilibrium molecular dynamics method. We have accurately tested the convergence of the simulation results for the thermal conductivity coefficient with respect to various simulation parameters. As far as we know, such a thorough analysis of the NEMD method for the thermal conductivity of 2D Yukawa systems in the presence of an external magnetic field had not been performed previously. Our findings reveal interesting insights into how the magnetic field affects the thermal conductivity of the system. The presence of a magnetic field leads to a significant decrease in the thermal conductivity coefficient. This decrease is caused by the rotation of particles around magnetic field lines, which leads to poorer energy transfer in the system. We found that weak coupling results in stronger sensitivity of the thermal conductivity on the external magnetic field effect. In the case of the strongly coupled particles, the electrostatic interaction-induced localisation of particles reduces the sensitivity of the thermal conduction on the effect of the external magnetic field.

This comprehensive numerical study contributes to our understanding of energy transfer in two-dimensional Yukawa systems, especially in the context of magnetic fields and plasma coupling parameters. The presented simulation results for the thermal conductivity of the 2D Yukawa system can be used to further develop various theoretical models for the thermal conductivity coefficient^31^.

## Data Availability

The datasets used and/or analysed during the current study available from the corresponding author on reasonable request.
